# Supplementary material for: Effects of optogenetic silencing the anterior cingulate cortex in a delayed non-match to trajectory task
Source: Oxf Open Neurosci. 2024 Feb 8;3:kvae002. doi: 10.1093/oons/kvae002 (PMC10939314; doi:10.1093/oons/kvae002)
Supplement: Web_Material_kvae002 [file Web_Material_kvae002.zip › SUPPLEMENTARY_MATERIALS_rev.pdf]

SUPPLEMENTARY MATERIAL

SUPPLEMENTARY FIGURES 1:3 and Tables 5:6

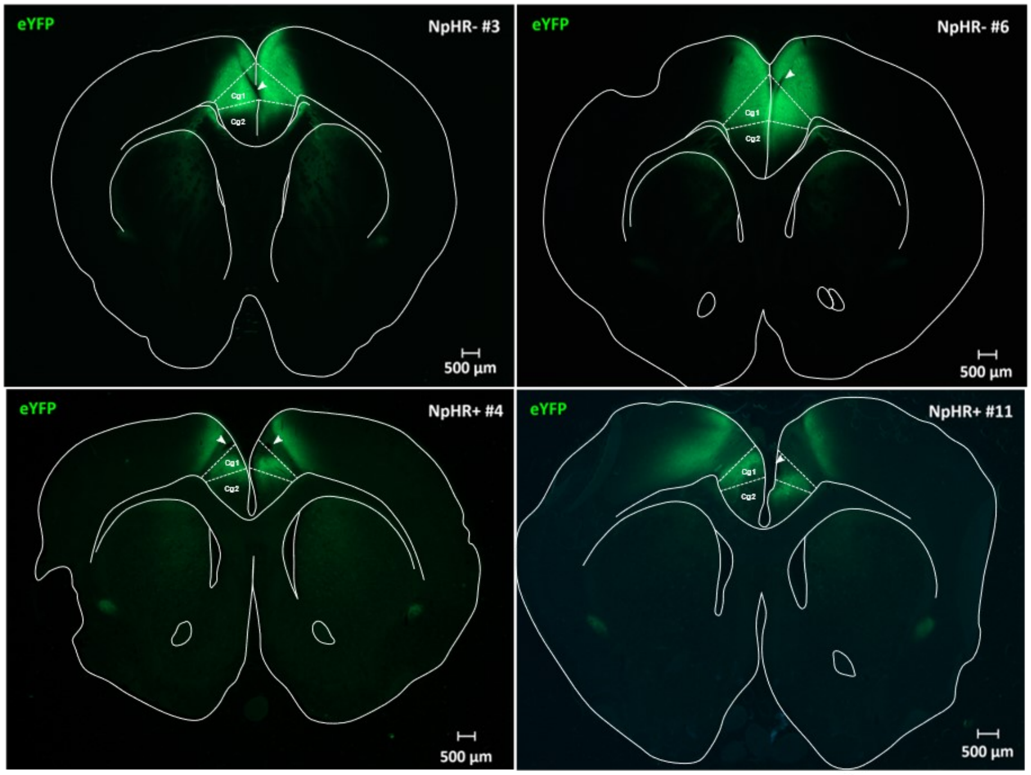

Figure S1. Histological verification of CTRLeYFP or eYFP expression after bilateral injection of viral constructs. (A) Examples eYFP expression and fiber entry lesion (arrow) in CTRL rats. (B) Similar to previous but for eYFP in eNpHR3.0+ rats: with two (left) or only one (right) fibers implanted. CG regions were outlined as in Paxinos Watson Rat Brain Atlas, at +1 A/P.

A

B

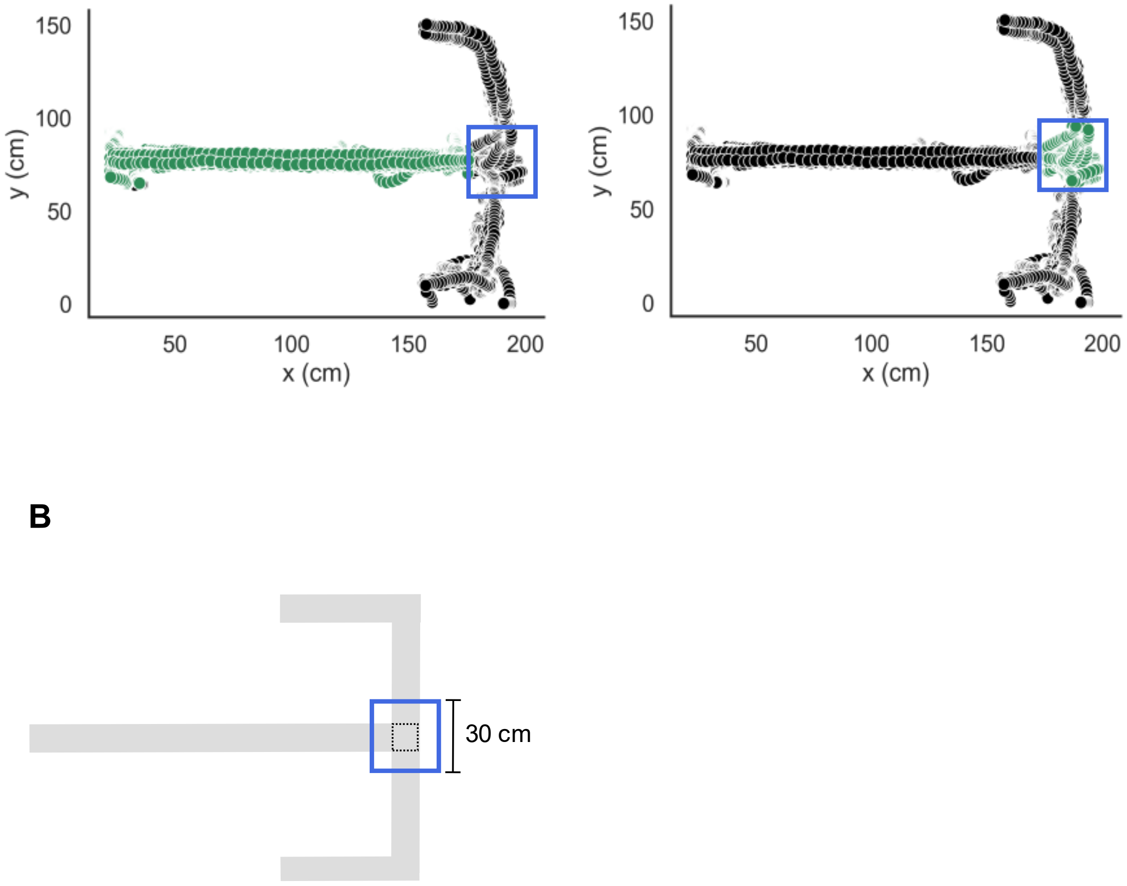

Figure S2. Measurement of latency to choice point and time spent in choice point. (A) Example position data collected from one session. Left, the position data before entering the choice point is colored green. Right, the position data inside the choice point is colored green. Latencies were computed from the timestamps corresponding to each data point series. (B). Scheme of the choice area used for latency calculations (in blue) corresponding to and the actual maze choice point (black square) plus 10 cm around.

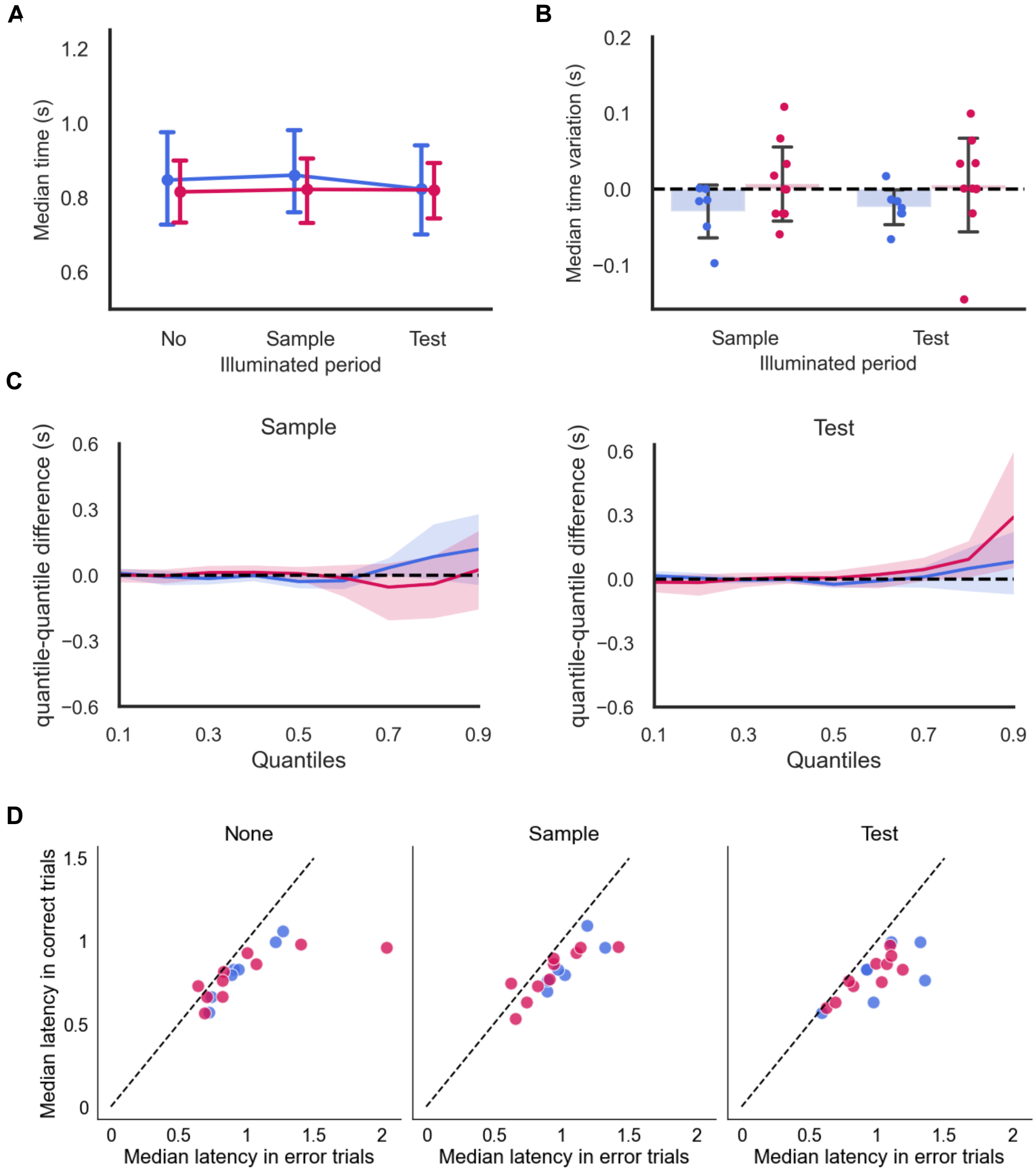

Figure S3. Time spent at the choice point. (A). Latency at the choice point medians across groups and manipulation types (CTRL: None: 0.84±0.18 s. Sample: 0.86±0.14 s. Test: 0.82±0.17 s. eNpHR3.0+: None: 0.82±0.15 s. Sample: 0.82±0.15 s. Test: 0.82±0.13 s). (B) Latency variation from non-illuminated trials, in seconds. The bars represent the average within-animal median difference (mean+/-SD), and the dots represent median latency differences within each animal (median illuminated - median non-illuminated) (CTRL: Sample: -0.029±0.038 s. Test -:0.024±0.025 s. eNpHR3.0+: Sample: 0.006±0.051 s Test: 0.005±0.065 s). (C) Similar to (B) but across all quantiles of the distributions, in seconds, for the sample illuminated trials (left) and test illuminated trials (right) (mean± CI(95%)) (D) Comparison between the median time in choice point on error vs. correct trials. Each dot corresponds to one rat. The dashed line corresponds to a situation in which both medians (in correct and error trials) have the same value. All summary data depicted is mean+/-SD.

Table5. Fixed Effects parameter estimates on time in choice point during experimental protocol

**Function**	Time to choice point ~ [1 + group x illumination x session number x trial number x outcome (1 + illumination| rat)]						
**Names**	**Effect**	**Estimate**	**SE**	**CI_L**	**CI_U**	**z**	***p***
(Intercept)	(Intercept)	0.94526	0.057	0.83363	1.05688	16.5975	< .001
illumination1	1 - 0	-0.00123	0.0284	-0.05688	0.05442	-0.0432	0.966
illumination2	3 - 0	-0.05287	0.0307	-0.11304	0.0073	-1.7223	0.085
outcome1	1 - 0	0.20722	0.0322	0.14412	0.27032	6.4365	< .001
trial number	trial number	0.07582	0.0161	0.04433	0.1073	4.7193	< .001
group1	NPHR - CTRL	-0.0148	0.1138	-0.23785	0.20826	-0.13	0.897
session number	session number	0.02849	0.0193	-0.00936	0.06634	1.4754	0.14
illumination1 ✻ outcome1	1 - 0 ✻ 1 - 0	-0.12952	0.0338	-0.19577	-0.06328	-3.8323	< .001
illumination2 ✻ outcome1	3 - 0 ✻ 1 - 0	-0.04021	0.0314	-0.10178	0.02136	-1.28	0.201
illumination1 ✻ group1	1 - 0 ✻ NPHR - CTRL	0.02419	0.0564	-0.08637	0.13475	0.4288	0.668
illumination2 ✻ group1	3 - 0 ✻ NPHR - CTRL	-0.02203	0.0612	-0.14196	0.0979	-0.36	0.719
outcome1 ✻ group1	1 - 0 ✻ NPHR - CTRL	-0.06462	0.0642	-0.19047	0.06123	-1.0064	0.314
illumination1 ✻ trial number	1 - 0 ✻ trial number	-0.02074	0.0134	-0.04697	0.00549	-1.5499	0.121
illumination2 ✻ trial number	3 - 0 ✻ trial number	-0.01898	0.0131	-0.04475	0.00678	-1.444	0.149
illumination1 ✻ session number	1 - 0 ✻ session number	0.01224	0.0139	-0.01498	0.03946	0.8812	0.378
illumination2 ✻ session number	3 - 0 ✻ session number	-0.00692	0.0135	-0.03332	0.01948	-0.5137	0.607
trial number ✻ group1	trial number ✻ NPHR - CTRL	-0.03035	0.032	-0.09316	0.03245	-0.9472	0.344
group1 ✻ session number	NPHR - CTRL ✻ session number	-0.01123	0.0383	-0.08627	0.06382	-0.2933	0.769

illumination1 ✻ outcome1 ✻ group1	1 - 0 ✻ 1 - 0 ✻ NPHR - CTRL	-0.1136	0.0676	-0.24607	0.01887	-1.6807	0.093
illumination2 ✻ outcome1 ✻ group1	3 - 0 ✻ 1 - 0 ✻ NPHR - CTRL	-0.03635	0.0628	-0.15949	0.0868	-0.5785	0.563
illumination1 ✻ trial number ✻ group1	1 - 0 ✻ trial number ✻ NPHR - CTRL	-0.00351	0.0267	-0.05594	0.04891	-0.1314	0.895
illumination2 ✻ trial number ✻ group1	3 - 0 ✻ trial number ✻ NPHR - CTRL	-0.06877	0.0263	-0.12023	-0.01731	-2.6193	0.090
illumination1 ✻ group1 ✻ session number	1 - 0 ✻ NPHR - CTRL ✻ session number	-0.02639	0.0278	-0.08087	0.02809	-0.9494	0.342
illumination2 ✻ group1 ✻ session number	3 - 0 ✻ NPHR - CTRL ✻ session number	-0.01898	0.0269	-0.07179	0.03382	-0.7046	0.481

Table6. Fixed Effect Omnibus test results using estimates from Table 1 in time to choice point during experimental protocol

Interaction	χ2	df	p
illumination	3.5049	2	0.173
outcome	41.4281	1	< .001
trial number	22.2721	1	< .001
group	0.0169	1	0.897
session number	2.1767	1	0.14
illumination ✻ outcome	15.2334	2	< .001
illumination ✻ group	0.5352	2	0.765
outcome ✻ group	1.0128	1	0.314

illumination ✻ trial number	3.0025	2	0.223
illumination ✻ session number	1.9824	2	0.371
group ✻ trial number	0.8972	1	0.344
group ✻ session number	0.086	1	0.769
illumination ✻ group ✻ outcome	2.9162	2	0.233
group ✻ trial number ✻ illumination	3.6825	2	0.183
illumination ✻ group ✻ session number	0.9721	2	0.615
