# Supplementary material for: Effects of optogenetic silencing the anterior cingulate cortex in a delayed non-match to trajectory task
Source: Oxf Open Neurosci. 2024 Feb 8;3:kvae002. doi: 10.1093/oons/kvae002 (PMC10939314; doi:10.1093/oons/kvae002)
Supplement: Web_Material_kvae002 [file Web_Material_kvae002.zip › Reviewers comments - Supplementary Material - OXFNSC-2023-016.R3 .docx]

**REVIEWER COMMENTS**

**OXFNSC-2023-016.R3 - Effects of optogenetic silencing the Anterior Cingulate Cortex in a delayed non-match to trajectory task**

**Original submission – OXFNSC-2023-016**

**Reviewer 1**

Comments to the Author

This manuscript describes a straightforward experiment that tested the necessity of the CG in working memory in rats. Using an optogenetic approach, the CG was silenced during the sample or test phase of a delayed nonmatch to position task. The trial types were interleaved within the session and a null virus control group was used. These were strengths of the experimental design. The weaknesses and/or points for clarification are listed below.

The statistical analysis decisions need to be clarified, especially the decision to use a t-test to analyze the possible difference between groups for the pre-manipulation testing and the decision to use a completely different statistical approach for the manipulation session. It is much more common to use ANOVAs and test for group x illumination condition interactions.

For the data shown in Figure 3 E and F, the effect sizes look very small. Would these results be statistically significant if the authors had used more conventional statistical tests?

What was the pretraining regimen for the rats?

I find it surprising that the ITI was shorter than the delay. Most studies that use the delayed nonmatch to position task make the ITI at least double the duration of the delay so that the rat is able to distinguish between trials/trial phases.

Was there a prediction about the differences in choice accuracy under sample vs. test illumination? If so, how does that prediction fit in to the theoretical model presented in Intro about the role of CG

How many rats were in each group?

Why were only male rats used?

FIg. 1 caption does not adequately describe what is being shown in the Figure.

Figs 3 and 4 should have a legend so the reader can tell what group corresponds to which color without having to read the figure caption.

**Reviewer 2**

Comments to the Author

The authors claim that inhibition of neurons in the anterior cingulate cortex of rats during the choice phase of a spatial working memory task reduces performance and shortens the delays for reaching a decision ("hastier decison-making"). The study is experimentally well conducted and important, but I do not agree with the conclusions as they do not appear to be supported by the data:

A) Fig. 3 and related Results section:

(a) Colour legend is missing in the figure.

(b) Panels C and D are not properly explained in the main text - what is shown here and why is it relevant?

(c) It is weird to compare TI to SI trials, as this is not related to the overall hypothesis of the experiment; the common control for both of them are the NI trials, so the statistics should be presented for the NI-vs-SI and NI-vs-TI comparisons.

(d) The fact that there is no between-subject difference in either SI or TI trials despite equal performance of both groups in NI trials is concerncing and considerably weaens the overall argument of an induced impairment.

(e) The authors only cite the main effect of TI and the post-hoc comparison, but the decisive question here is if there is phase(NI-TI)-group interaction (which I doubt), only then it can be solidly claimed that optical stimulation affects the NpHR group selectively (i.e. not the control group).

(f) The value of panel F is unclear to me; it rather underlines the notion that TI leads to mildly decreased performance, numerically speaking, in both groups, just that the - again: not really relevant - comparison to SI is borderline non-significant in the controls and borderline-significant in the eNpHR animals. If data is presented as such, the proper analysis in my view would be a one-sample test against 0 for all 4 conditions. Judging from the distribution and variance there is either no impairment or a mild impairment in both groups. Also, the displayed value does not seem to be a "performance variation"-metric, but simply a baseline-normalized performance, so the axis is misleading. The authors could check if log-transformation, which would be a recommended measure for ratios before applying parametric testing yields values with which the one-sample t-test becomes significant.

B) Fig 4 and related Results section:

My basic criticism is the same as for Fig. 3 ...

(a) There are no between-subject differences.

(b) Within-subject comparisons need to be made with NI-TI and NI-SI comparisons to be meaningful, not with SI-TI comparisons.

(c) Standard repeated measures ANOVAs for absolute values and one-sample t-tests or between-subject paried t-tests for NI-baseline-normalized values would help to clarify selective effects in just one group (for Fig. 4, the control group)

(d) The explanation "The opposite happens with the betterperforming CTRL animals" is not logically sound - why should the control animals require more decision time compared to their own NI baseline condition or compared to SI, where they perform apparantly equally well, according to the interpretation of the authors. Also, the sentence implies a between-subject difference in performance, which does not exist.

(e) Importantly, the data is entirely misrepresented in the Abstract of the manuscript, in my view, which states that CG-inhibition leads to shorter delays / hastier decision-making. Numerically even the opposite is true, the decision latency is longer in TI compared to SI and NI. And statistically, there is simply no effect on latencies in the NpHR-group.

C) I disagree with the overall conclusion from Fig. 3 and from the paper that the mildly reduced performance with TI implies a significant role of the CG in working memory. The weakness of the effect that is only statistically apparant in a within-subject, not a between-subject comparison, and possibly not even in the most relevant comparison (TI vs NI), is more in line with an indirect effect on the WM performance metric; for example, the CG has a role in visual attentional control (see e.g. http://www.nature.com/npp/journal/v41/n4/abs/npp2015229a.html , https://www.sciencedirect.com/science/article/pii/S0896627321000751 , https://www.nature.com/articles/s41398-023-02541-z ) and interference with that would also impact on performace in a spatial working memory task without affecting working memory as such. Since attention and other decision-making variables cannot be controlled for in this task, the conclusion needs to moderated down, in any case - but more likely inversed to concluding a negative effect: The combined view of Fig. 3 and Fig. 4, esp. 3F and 4B, rather suggests that both groups are mildly affected by TI-stimulation, speaking for an unspecific effect of warming of the tissue (which occurs with optogenetic stimulation) or a distraction occuring right at the time when the decision needs to be made (hence no effect in SI) - this mildly/numerically reduces performance and increases latency, and these effects, which are both numerically present in BOTH groups, reach significance for performance in the NpHR group and for latency in the control group.

I think overall the modified manuscript could be published, but as a document of a negative result of ACC stimulation on working memory, evidenced by lack of between-subject differences and possibly even lack of significant NI-TI within-subject differences, when applying standard statistical tests. The current conclusion seems to be misleading to me, as it is not supported by the presented data. As stated above (B(e)), the conclusion regarding effects on delays presented in the Abstract is entirely false and misleading in light of the presented data.

I also have a few minor comments:

1) The expression pattern in the eNpHR group seems to be limited to the ventral ACC (Cg2) and the ACC/M2 border (Fig. 1). Can the authors comment if this was generally the case or if dorsal ACC (Cg1) was also targeted in a significant proportion of animals.

2) While the authors are correct that optogenetic inhibition studies in WM tasks have not been pulblished yet, a strong indication of the ACC involvement in WM comes from the observation of delay-activity of ACC cells in monkeys, which was not mentioned in the manuscript:

Niki H, Watanabe M. Cingulate unit activity and delayed response. Brain Res. 1976;110: 381–386. doi:10.1016/0006-8993(76)90412-1

Niki H, Watanabe M. Prefrontal and cingulate unit activity during timing behavior in the monkey. Brain Res. 1979;171: 213–224. doi:10.1016/0006-8993(79)90328-7

3) The subpanels of Figure 2C-F are not properly referenced, making it difficult to match the description of page 5 with the data figures. Esp. the difference evident in 2F needs some explanation.

4) Please state in the Results, p. 5-6, which delay times were used for training and testing (with optogenetic modulation).

**Decision letter**

19-Oct-2023

Dear Dr. Remondes,

Manuscript ID OXFNSC-2023-016 entitled "Optogenetic silencing of the Anterior Cingulate Cortex impairs working memory in a delayed non-match to trajectory task" which you submitted to the Oxford Open Neuroscience, has been seen by two reviewers with relevant expertise. The comments of the reviewer(s) are included at the bottom of this letter. I have also read it myself.

Both reviewers praised the experimental design and agree that this work is potentially important. However, they also have serious concerns about the statistical approach you have used and the justification for some of the key conclusions.

A central issue raised by both reviewers is that you have drawn conclusions about silencing of CG without reporting a direct statistical evaluation of the group difference. You say that silencing CG 'significantly decreases' WM performance, both in the abstract and the discussion. This implies a significant difference between the eNpHR3.0+ group (in which CG was silenced) and the control group (in which it was not). But the analysis reported on page 6 only shows a significant effect of TI versus SI condition (across both groups) along with the finding that this effect was significant in the eNpHR3.0+ group (p=0.032) but not the control group (no statistics reported). Finding that an effect is significant in one group without being significant in the other does not provide a sufficient basis for claiming a group difference, or implying that the group difference itself is significant.

I am therefore rejecting this version of the manuscript, however I would like to invite you to submit a revised manuscript if you are willing to amend the manner in which you interpret your results and draw conclusions (or explain why the statistical concerns are mistaken). As noted above, both reviewers praised the experimental design and suggest that your work could make an important contribution, potentially as a report of a null or inconclusive effect if the statistical basis for claiming an impact of CG silencing is lacking.

Both reviewers have made several other helpful comments which you should also consider in preparing a revision.

Please note that this journal operates with transparent peer review. This means that if your submission is accepted for publication, the full peer review history of your article will publish online alongside your article. This includes reviewer comments, editor decision letters, and your author responses.

I invite you to respond to the reviewer(s)' comments and revise your manuscript within the next 60 days from today date. Please contact the editorial office if you require a longer period.

To revise your manuscript, log into https://mc.manuscriptcentral.com/oxfnsc and enter your Author Centre, where you will find your manuscript title listed under "Manuscripts with Decisions." Under "Actions," click on "Create a Revision." Your manuscript number has been appended to denote a revision.

You may also click the below link to start the revision process (or continue the process if you have already started your revision) for your manuscript. If you use the below link you will not be required to login to ScholarOne Manuscripts.

*** PLEASE NOTE: This is a two-step process. After clicking on the link, you will be directed to a webpage to confirm. ***

https://mc.manuscriptcentral.com/oxfnsc?URL_MASK=a7d15f3fe88f4c8093683163e1da9a27

You will be unable to make your revisions on the originally submitted version of the manuscript. Instead, revise your manuscript using a word processing program and save it on your computer. Please also highlight the changes to your manuscript within the document by using the track changes mode in MS Word or by using bold or colored text.

Once the revised manuscript is prepared, you can upload it and submit it through your Author Centre.

When submitting your revised manuscript, you will be able to respond to the comments made by the reviewer(s) in the space provided. You can use this space to document any changes you make to the original manuscript. In order to expedite the processing of the revised manuscript, please be as specific as possible in your response to the reviewer(s).

IMPORTANT: Your original files are available to you when you upload your revised manuscript. Please delete any redundant files before completing the submission.

Once again, thank you for submitting your manuscript to the Oxford Open Neuroscience and I look forward to receiving your revision.

Sincerely,

Professor Sam Gilbert

Senior Editor, Oxford Open Neuroscience

sam.gilbert@ucl.ac.uk

Reviewer: 1

Comments to the Author

This manuscript describes a straightforward experiment that tested the necessity of the CG in working memory in rats. Using an optogenetic approach, the CG was silenced during the sample or test phase of a delayed nonmatch to position task. The trial types were interleaved within the session and a null virus control group was used. These were strengths of the experimental design. The weaknesses and/or points for clarification are listed below.

The statistical analysis decisions need to be clarified, especially the decision to use a t-test to analyze the possible difference between groups for the pre-manipulation testing and the decision to use a completely different statistical approach for the manipulation session. It is much more common to use ANOVAs and test for group x illumination condition interactions.

For the data shown in Figure 3 E and F, the effect sizes look very small. Would these results be statistically significant if the authors had used more conventional statistical tests?

What was the pretraining regimen for the rats?

I find it surprising that the ITI was shorter than the delay. Most studies that use the delayed nonmatch to position task make the ITI at least double the duration of the delay so that the rat is able to distinguish between trials/trial phases.

Was there a prediction about the differences in choice accuracy under sample vs. test illumination? If so, how does that prediction fit in to the theoretical model presented in Intro about the role of CG

How many rats were in each group?

Why were only male rats used?

FIg. 1 caption does not adequately describe what is being shown in the Figure.

Figs 3 and 4 should have a legend so the reader can tell what group corresponds to which color without having to read the figure caption.

Reviewer: 2

Comments to the Author

The authors claim that inhibition of neurons in the anterior cingulate cortex of rats during the choice phase of a spatial working memory task reduces performance and shortens the delays for reaching a decision ("hastier decison-making"). The study is experimentally well conducted and important, but I do not agree with the conclusions as they do not appear to be supported by the data:

A) Fig. 3 and related Results section:

(a) Colour legend is missing in the figure.

(b) Panels C and D are not properly explained in the main text - what is shown here and why is it relevant?

(c) It is weird to compare TI to SI trials, as this is not related to the overall hypothesis of the experiment; the common control for both of them are the NI trials, so the statistics should be presented for the NI-vs-SI and NI-vs-TI comparisons.

(d) The fact that there is no between-subject difference in either SI or TI trials despite equal performance of both groups in NI trials is concerncing and considerably weaens the overall argument of an induced impairment.

(e) The authors only cite the main effect of TI and the post-hoc comparison, but the decisive question here is if there is phase(NI-TI)-group interaction (which I doubt), only then it can be solidly claimed that optical stimulation affects the NpHR group selectively (i.e. not the control group).

(f) The value of panel F is unclear to me; it rather underlines the notion that TI leads to mildly decreased performance, numerically speaking, in both groups, just that the - again: not really relevant - comparison to SI is borderline non-significant in the controls and borderline-significant in the eNpHR animals. If data is presented as such, the proper analysis in my view would be a one-sample test against 0 for all 4 conditions. Judging from the distribution and variance there is either no impairment or a mild impairment in both groups. Also, the displayed value does not seem to be a "performance variation"-metric, but simply a baseline-normalized performance, so the axis is misleading. The authors could check if log-transformation, which would be a recommended measure for ratios before applying parametric testing yields values with which the one-sample t-test becomes significant.

B) Fig 4 and related Results section:

My basic criticism is the same as for Fig. 3 ...

(a) There are no between-subject differences.

(b) Within-subject comparisons need to be made with NI-TI and NI-SI comparisons to be meaningful, not with SI-TI comparisons.

(c) Standard repeated measures ANOVAs for absolute values and one-sample t-tests or between-subject paried t-tests for NI-baseline-normalized values would help to clarify selective effects in just one group (for Fig. 4, the control group)

(d) The explanation "The opposite happens with the betterperforming CTRL animals" is not logically sound - why should the control animals require more decision time compared to their own NI baseline condition or compared to SI, where they perform apparantly equally well, according to the interpretation of the authors. Also, the sentence implies a between-subject difference in performance, which does not exist.

(e) Importantly, the data is entirely misrepresented in the Abstract of the manuscript, in my view, which states that CG-inhibition leads to shorter delays / hastier decision-making. Numerically even the opposite is true, the decision latency is longer in TI compared to SI and NI. And statistically, there is simply no effect on latencies in the NpHR-group.

C) I disagree with the overall conclusion from Fig. 3 and from the paper that the mildly reduced performance with TI implies a significant role of the CG in working memory. The weakness of the effect that is only statistically apparant in a within-subject, not a between-subject comparison, and possibly not even in the most relevant comparison (TI vs NI), is more in line with an indirect effect on the WM performance metric; for example, the CG has a role in visual attentional control (see e.g. http://www.nature.com/npp/journal/v41/n4/abs/npp2015229a.html , https://www.sciencedirect.com/science/article/pii/S0896627321000751 , https://www.nature.com/articles/s41398-023-02541-z ) and interference with that would also impact on performace in a spatial working memory task without affecting working memory as such. Since attention and other decision-making variables cannot be controlled for in this task, the conclusion needs to moderated down, in any case - but more likely inversed to concluding a negative effect: The combined view of Fig. 3 and Fig. 4, esp. 3F and 4B, rather suggests that both groups are mildly affected by TI-stimulation, speaking for an unspecific effect of warming of the tissue (which occurs with optogenetic stimulation) or a distraction occuring right at the time when the decision needs to be made (hence no effect in SI) - this mildly/numerically reduces performance and increases latency, and these effects, which are both numerically present in BOTH groups, reach significance for performance in the NpHR group and for latency in the control group.

I think overall the modified manuscript could be published, but as a document of a negative result of ACC stimulation on working memory, evidenced by lack of between-subject differences and possibly even lack of significant NI-TI within-subject differences, when applying standard statistical tests. The current conclusion seems to be misleading to me, as it is not supported by the presented data. As stated above (B(e)), the conclusion regarding effects on delays presented in the Abstract is entirely false and misleading in light of the presented data.

I also have a few minor comments:

1) The expression pattern in the eNpHR group seems to be limited to the ventral ACC (Cg2) and the ACC/M2 border (Fig. 1). Can the authors comment if this was generally the case or if dorsal ACC (Cg1) was also targeted in a significant proportion of animals.

2) While the authors are correct that optogenetic inhibition studies in WM tasks have not been pulblished yet, a strong indication of the ACC involvement in WM comes from the observation of delay-activity of ACC cells in monkeys, which was not mentioned in the manuscript:

Niki H, Watanabe M. Cingulate unit activity and delayed response. Brain Res. 1976;110: 381–386. doi:10.1016/0006-8993(76)90412-1

Niki H, Watanabe M. Prefrontal and cingulate unit activity during timing behavior in the monkey. Brain Res. 1979;171: 213–224. doi:10.1016/0006-8993(79)90328-7

3) The subpanels of Figure 2C-F are not properly referenced, making it difficult to match the description of page 5 with the data figures. Esp. the difference evident in 2F needs some explanation.

4) Please state in the Results, p. 5-6, which delay times were used for training and testing (with optogenetic modulation).

**Author response**

Lisbon, November 9th, 2023

Dear Dr Gilbert,

We thank you for your reply, for the time taken to read, and for the chance to resubmit, our manuscript ID OXFNSC-2023-016, considering your impressions, the Reviewers’ criticisms and highly valuable advice, and our own reassessment of the data considering that input.

We have now agreed that all the concerns presented are justified, as our data do not support the claims made in their presented form. We do not see a performance difference between eNpHR3.0+ and CTRL groups, only performance differences within the former group depending on when light is delivered, and equivalent differences in the dynamic of behaviour. As such, rather than evidence of an absolute impairment in performance, we now see this as suggesting an interference with more subtle aspects of the neural mechanisms supporting memory-based decision, warranting further studies rather than an explicit conclusion.

As a result of our reassessment, we have now substantially changed the claims made before, and revised the manuscript accordingly. In our revision we replied to all the major and minor comments made by the Reviewers. We have now provided a point-by-point reply to these comments, for the sake of time abstaining from a detailed description of changes made, which we find perhaps redundant, but one we’re willing to provide if found necessary.

We hope you find the present manuscript worthy of publication in OON.

Please let us know should further questions arise.

Best regards

Miguel

**Decision letter - OXFNSC-2023-016.R1**

08-Dec-2023

Dear Dr. Remondes,

Thank you for your revised manuscript OXFNSC-2023-016.R1 entitled "Effects of optogenetic silencing the Anterior Cingulate Cortex in a delayed non-match to trajectory task" which you submitted to the Oxford Open Neuroscience.

Your manuscript has been re-reviewed by the original two reviewers and I have also read it myself.

As you will see, the reviewers no longer have serious concerns about the overall statistical approach and the way that you interpret results. However, reviewer 2 requests some additional statistical tests (as initially suggested as well by reviewer 1 in the first round of reviews).

I agree with the reviewers that additional ANOVAs would help the reader interpret the results. Therefore I am inviting you to submit a revised manuscript with the following additions:

1. As suggested by reviewer 2, please report results from two mixed 2 x 2 ANOVAs. The first should include factors Phase (NI, TI) and Group (eNpHR3.0+, Control). The second should include Phase (NI, SI) and Group (eNpHR3.0+, Control).

Considering that you have also reported baseline-normalised scores (NI-SI, NI-TI), you might also consider a 2 x 2 ANOVA with factors Phase (baseline-normalised-SI, baseline-normalised-TI) and Group (eNpHR3.0+, Control). But I will leave this up to you.

2. One of your key conclusions rests on a significant t test for the NI vs TI comparison in the eNpHR3.0+ group but not the Control group. However, you do not report any statistical test for the Control group, only that it was nonsignificant. Please report the results of these statistical tests in full.

3. Figure 3F shows the results from your baseline-normalised performance measure, indicating whether or not each test was significant but no further detail. Please also report these statistical tests in full.

Alternatively, if you think that these tests are not appropriate, please explain why.

I will aim to deal with any revised manuscript swiftly, without further review.

Please note that this journal operates with transparent peer review. This means that if your submission is accepted for publication, the full peer review history of your article will publish online alongside your article. This includes reviewer comments, editor decision letters, and your author responses.

To revise your manuscript, log into https://mc.manuscriptcentral.com/oxfnsc and enter your Author Centre, where you will find your manuscript title listed under "Manuscripts with Decisions." Under "Actions," click on "Create a Revision." Your manuscript number has been appended to denote a revision.

You may also click the below link to start the revision process (or continue the process if you have already started your revision) for your manuscript. If you use the below link you will not be required to login to ScholarOne Manuscripts.

*** PLEASE NOTE: This is a two-step process. After clicking on the link, you will be directed to a webpage to confirm. ***

https://mc.manuscriptcentral.com/oxfnsc?URL_MASK=bdbf027e29c044bcb6225f547612b827

You will be unable to make your revisions on the originally submitted version of the manuscript. Instead, revise your manuscript using a word processing program and save it on your computer. Please also highlight the changes to your manuscript within the document by using the track changes mode in MS Word or by using bold or colored text.

Once the revised manuscript is prepared, you can upload it and submit it through your Author Centre.

When submitting your revised manuscript, you will be able to respond to the comments made by the reviewer(s) in the space provided. You can use this space to document any changes you make to the original manuscript. In order to expedite the processing of the revised manuscript, please be as specific as possible in your response to the reviewer(s).

IMPORTANT: Your original files are available to you when you upload your revised manuscript. Please delete any redundant files before completing the submission.

Because we are trying to facilitate timely publication of manuscripts submitted to the Oxford Open Neuroscience, your revised manuscript should be uploaded within the next 30 days from todays date. If it is not possible for you to submit your revision in a reasonable amount of time, we may have to consider your paper as a new submission.

Once again, thank you for submitting your manuscript to the Oxford Open Neuroscience and I look forward to receiving your revision.

Sincerely,

Dr. Sam Gilbert

Senior Editor, Oxford Open Neuroscience

sam.gilbert@ucl.ac.uk

Reviewer: 2

Comments to the Author

The revised interpretation of the data is now largely sound. I - just like Reviewer 1, in the first round of revision - would nevertheless suggest to report phase-group interaction effects and main effects of 2-way ANOVAs (for NI-SI and NI-TI x group) for performance and latencies in order to be able to evaluate to what extent effects of the light-stimulation are specific or not specific. Also, I assume the title is lacking an "of" after "silencing"?

Reviewer: 1

Comments to the Author

Many of my comments were not addressed. Instead, they were dismissed and/or just addressed in the Author's response and not addressed in the manuscript itself.

Associate Editor

Comments to the Author:

(There are no comments.)

**Author response - OXFNSC-2023-016.R1**

Lisbon, December 26th, 2023

Dear Dr Gilbert,

We are, once again, quite thankful for the time both you and the Reviewers have taken to read and critically evaluate our re-submitted manuscript, thus contributing in a fundamental way for its improvement into, what we hope is, a version of record. We are glad that we have now provided a satisfactory reply to the Reviewer’s concerns and criticisms.

Concerning your final request of providing a full description of the results of proposed statistical tests, we have now provided all the required statistical reporting, based on the experimental and analytical design we have defined a priori. In addition to these changes, and after careful consideration of your last communication vis-à-vis our experimental and analytical planning, and our convictions, we have decided to take your second suggestion:

“Alternatively, if you think that these tests are not appropriate, please explain why”.

We will, therefore, now proceed to explain why we do not consider the proposed t-tests and ANOVA the most appropriate analyses for the data at hand. A useful reference to support our approach can be found in (Yu et al., 2021).

The experiments we now report were designed together with the statistical comparisons already in mind, given the fact that they include distinct types of data, unknown underlying distributions, multiple subjects and measurements within subjects, multiple experimental groups across subject groups, data groups that were explicitly not independent and that had non-homogeneous variances. Such properties as a whole preclude the use of the proposed ANOVA. Furthermore, even if the data were normally distributed - which it isn’t and this is obvious for latencies - separating the levels within factors as proposed in the suggested ANOVAs is also not the most advisable practice, as it isn’t to run repeated distinct tests on already analyzed data. Finally, since the design was chosen taking the data into account, the model used at each stage of analysis was selected to closely match (quantitatively) the distributions underlying the sampled response variables, rather than some arbitrarily chosen distribution as it is the case in ANOVA.

In our research group we routinely prefer to use Generalized Linear Mixed Models (GLMMs) over traditional ANOVA and other parametric tests. Here are some reasons why:

1. GLMMs can handle various types of response variables (continuous, binary, and so on) and are not limited to the assumptions of normality, and homogeneity of variance, required by ANOVA.

2. GLMMs allow the inclusion of random effects, accounting for hierarchical or clustered data structures, as is the case in the present datasets, and accommodating such factors as trial number and session number, which we included. As already mentioned, ANOVA usually assumes independence among observations, which in our data is explicitly not the case.

3. ANOVA relies on the assumption of normally distributed residuals, something not met in our data - in fact, latencies exhibit a heavily skewed distribution. Additionally, we are dealing with different types of variables, distributed differently.

4. As alluded to earlier, GLMMs have greater statistical power compared to ANOVA, especially when dealing with our type of datasets, inherently complex and mixed.

5. The methodology we opted for allows us to incorporate all existing fixed and random effects simultaneously, and thus it allows a more sophisticated modeling of the data vis-à-vis the “real” distributions underlying the samples we ended up with, especially if compared to the proposed approach – ANOVA – whose approach is just too simple.

As to the following suggestion:

“1. As suggested by reviewer 2, please report results from two mixed 2 x 2 ANOVAs. The first should include factors Phase (NI, TI) and Group (eNpHR3.0+, Control). The second should include Phase (NI, SI) and Group (eNpHR3.0+, Control).”

We believe that running two ANOVA to separately test distinct levels within the same factor, as proposed here is not advisable for the following theoretical reasons:

1. When conducting multiple pairwise comparisons between levels within the same factor, the likelihood of making a Type I error (false positive) increases.

2. Since ANOVA tests differences among groups within a factor, it must consider the whole variance across all groups, something that separating levels, as proposed, does not.

3. Isolating levels precludes the testing of all possible interactions in the whole context of the experiment, leading to oversimplifications, and not capturing important differences.

Finally, GLMMs in general, and our analysis in particular, improve statistical power, which is desirable for several reasons:

1. Increased probability of detecting true effects, minimizing the chances of missing real ones (Type II error).

2. Less waste of resources, as we take full advantage of human effort, time, and money.

3. Compliance with ethical regulations, because of point 2 above, and by rationalizing experimental design and animal numbers.

Two remaining points presented merit a separate response, which we now provide:

“2. One of your key conclusions rests on a significant t test for the NI vs TI comparison in the eNpHR3.0+ group but not the Control group. However, you do not report any statistical test for the Control group, only that it was nonsignificant. Please report the results of these statistical tests in full.”

Response: There were no t-tests, we reported the results of the post-hoc comparisons following GLMM testing, whose p-value led us to deem the difference not significant.

“3. Figure 3F shows the results from your baseline-normalised performance measure, indicating whether or not each test was significant but no further detail. Please also report these statistical tests in full.”

Response: The baseline-normalised performance plot was added to visually remove the effect of data variability. The asterisk refers to the previously reported result (3E), not to any testing done in these normalized data per se. We have now removed the asterisk from this plot.

It is our conviction that the course we took in our analysis is the correct one, considering the data and experimental design. We have so far accepted all the Reviewers’ advice and comments after careful consideration, something that transformed our manuscript in a way that, we firmly believe, much better reflects the results obtained. However, the same firmness now leads us to respectfully decline to perform the proposed analyses, and take your alternative suggestion by providing reasons we believe are clear enough for readers to understand why we chose the way we did.

We are aware of how much the Reviewers, and you as Editor, contributed to a fundamental leap in quality of our manuscript. And for this we thank you.

Best regards

Sofia and Miguel

REFERENCE CITED

Yu, Z., Guindani, M., Grieco, S.F., Chen, L., Holmes, T.C., and Xu, X. (2021). Beyond t test and ANOVA: applications of mixed-effects models for more rigorous statistical analysis in neuroscience research. Neuron. 10.1016/J.NEURON.2021.10.030

**Decision letter - OXFNSC-2023-016.R2**

09-Jan-2024

Dear Dr. Remondes,

Thank you for submitting Manuscript ID OXFNSC-2023-016.R2 entitled "Effects of optogenetic silencing the Anterior Cingulate Cortex in a delayed non-match to trajectory task".

I a grateful for the detailed response to the previous decision letter, which I have read and discussed with the editorial team at Oxford Open Neuroscience.

First of all, let me apologise for my inaccurate characterisation of your statistical approach in point 2 of my previous decision letter. The point I was trying to make is that you do not present enough statistical detail for a clear understanding of your results, but in making this point I incorrectly described the statistics that were presented. This was my error and I apologise for this. However, the point remains that the level of statistical detail is rather sparse.

I appreciate the points you make in favour of Generalized Linear Mixed Models in preference to other statistical approaches such as ANOVA. I do not wish to be prescriptive about exactly what statistical approach you use. Nevertheless, if you would prefer not to include the suggested analyses, I do think some further statistical detail is required.

I would therefore suggest you include additional information about the GLMM analyses that you have conducted. In particular, I suggest that you include both 1) the GLMM formula(s), and 2) a table with a summary of the GLMM analyses. For a recent example from Oxford Open Neuroscience that includes statistical reporting of this form, see Tables 1 and 2 from this paper:

https://academic.oup.com/oons/article/doi/10.1093/oons/kvad005/7087289

Alternatively, you might consider a link to data and code to reproduce your analyses on a repository such as https://osf.io.

I will look forward to receiving a revision that provides fuller statistical information. I will aim to deal with any revision swiftly.

Please note that this journal operates with transparent peer review. This means that if your submission is accepted for publication, the full peer review history of your article will publish online alongside your article. This includes reviewer comments, editor decision letters, and your author responses.

I invite you to revise your manuscript within the next 30 days from today's date.

To revise your manuscript, log into https://mc.manuscriptcentral.com/oxfnsc and enter your Author Centre, where you will find your manuscript title listed under "Manuscripts with Decisions." Under "Actions," click on "Create a Revision." Your manuscript number has been appended to denote a revision.

You may also click the below link to start the revision process (or continue the process if you have already started your revision) for your manuscript. If you use the below link you will not be required to login to ScholarOne Manuscripts.

*** PLEASE NOTE: This is a two-step process. After clicking on the link, you will be directed to a webpage to confirm. ***

https://mc.manuscriptcentral.com/oxfnsc?URL_MASK=0ac1e6917fa74017accd2fbc40ae985e

You will be unable to make your revisions on the originally submitted version of the manuscript. Instead, revise your manuscript using a word processing program and save it on your computer. Please also highlight the changes to your manuscript within the document by using the track changes mode in MS Word or by using bold or colored text.

Once the revised manuscript is prepared, you can upload it and submit it through your Author Centre.

When submitting your revised manuscript, you will be able to respond to the comments made by the reviewer(s) in the space provided. You can use this space to document any changes you make to the original manuscript. In order to expedite the processing of the revised manuscript, please be as specific as possible in your response to the reviewer(s).

IMPORTANT: Your original files are available to you when you upload your revised manuscript. Please delete any redundant files before completing the submission.

Because we are trying to facilitate timely publication of manuscripts submitted to the Oxford Open Neuroscience, your revised manuscript should be uploaded within the next 30 days from todays date. If it is not possible for you to submit your revision in a reasonable amount of time, we may have to consider your paper as a new submission.

Once again, thank you for submitting your manuscript to the Oxford Open Neuroscience and I look forward to receiving your revision.

Sincerely,

Professor Sam Gilbert

Senior Editor, Oxford Open Neuroscience

[sam.gilbert@ucl.ac.uk](mailto:sam.gilbert@ucl.ac.uk)

**Author response - OXFNSC-2023-016.R2**

Lisbon, January 16th, 2024

Dear Dr Gilbert,

Thank you for your kind reply. As per your request, we have now uploaded the required GLMM tables with the formulas as suggested. These are Tables 1-4. We have added references to these additional materials in the text and figure legends as appropriate.

We have also added Tables 5-6 to the Supplementary Materials, with the GLMM formulas corresponding to the plots therein.

We thank you and the Reviewers for the time taken to help us improve our manuscript.

Best regards

Sofia and Miguel

**Decision letter - OXFNSC-2023-016.R3**

19-Jan-2024

Dear Dr. Remondes,

It is a pleasure to accept your manuscript entitled "Effects of optogenetic silencing the Anterior Cingulate Cortex in a delayed non-match to trajectory task" in its current form for publication in Oxford Open Neuroscience.

Please note that this journal operates with transparent peer review. This means that the full peer review history of your article will publish online alongside your article. This includes reviewer comments, editor decision letters, and your author responses.

Next steps

You will receive an email from no-reply@scipris.com within roughly one week. This is your invitation to sign up for an account with SciPris, Oxford University Press’ author portal hosted by Aptara. You will need to create an account if you do not already hold one. Please register or log into your account and follow the online instructions which will guide you through signing your licence and paying the APC. The email and the portal have clearly signposted support options if you need any help during this process.

Please note that SciPris is a completely different system from ScholarOne, so your credentials to submit your manuscript here will not work there. Once you’ve created a SciPris account, you will be able to use it whenever you publish with Oxford Open Neuroscience or any OUP journal. Please note that OUP will only ever request payment for applicable fees be made via SciPris or to an OUP bank account. If you ever have concerns about the legitimacy of a request, please do not hesitate to contact a customer services agent via the SciPris portal or directly via oupsupport@scipris.com.

Thank you for your fine contribution. On behalf of the Editors of the Oxford Open Neuroscience, we look forward to your continued contributions to the Journal.

Sincerely,

Professor Sam Gilbert

Senior Editor, Oxford Open Neuroscience

sam.gilbert@ucl.ac.uk

Reviewer(s)' Comments to Author:
